# High-Resolution Simultaneous Multi-Slice Accelerated Turbo Spin-Echo Musculoskeletal Imaging: A Head-to-Head Comparison With Routine Turbo Spin-Echo Imaging

**DOI:** 10.3389/fphys.2021.759888

**Published:** 2021-12-21

**Authors:** Feifei Gao, Zejun Wen, Shewei Dou, Xiaojing Kan, Shufang Wei, Yinghui Ge

**Affiliations:** ^1^Department of Radiology, Henan Provincial People’s Hospital, Zhengzhou, China; ^2^Department of Radiology, West China Hospital, Sichuan University, Chengdu, China; ^3^Department of Radiology, Fuwai Central China Cardiovascular Hospital, Zhengzhou, China

**Keywords:** musculoskeletal imaging, turbo spin echo, simultaneous multi-slice, image quality, magnetic resonance imaging

## Abstract

**Background/Aim:** The turbo spin-echo (TSE) sequence is widely used for musculoskeletal (MSK) imaging; however, its acquisition speed is limited and can be easily affected by motion artifacts. We aimed to evaluate whether the use of a simultaneous multi-slice TSE (SMS-TSE) sequence can accelerate MSK imaging while maintaining image quality when compared with the routine TSE sequence.

**Methods:** We prospectively enrolled 71 patients [mean age, 37.43 ± 12.56 (range, 20–67) years], including 37 men and 34 women, to undergo TSE and SMS sequences. The total scanning times for the wrist, ankle and knee joint with routine sequence were 14.92, 13.97, and 13.48 min, respectively. For the SMS-TSE sequence, they were 7.52, 7.20, and 6.87 min. Quantitative parameters, including the signal-to-noise ratio (SNR) and contrast-to-noise ratio (CNR), were measured. Three experienced MSK imaging radiologists qualitatively evaluated the image quality of bone texture, cartilage, tendons, ligament, meniscus, and artifact using a 5-point evaluation system, and the diagnostic performance of the SMS-TSE sequences was evaluated.

**Results:** Compared with the routine TSE sequences, the scanning time was lower by 49.60, 48.46, and 49.04% using SMS-TSE sequences for the wrist, ankle, and knee joints, respectively. For the SNR comparison, the SMS-TSE sequences were significantly higher than the routine TSE sequence for wrist (except for Axial-T2WI-FS), ankle, and knee joint MR imaging (*all p* < 0.05), but no statistical significance was obtained for the CNR measurement (*all p* > 0.05, except for Sag-PDWI-FS in ankle joint). For the wrist joint, the diagnostic sensitivity, specificity, and accuracy were 88.24, 100, and 92%. For the ankle joint, they were 100, 75, and 93.33%. For the knee joint, they were 87.50, 85.71, and 87.10%.

**Conclusion:** The use of the SMS-TSE sequence in the wrist, ankle, and knee joints can significantly reduce the scanning time and show similar image quality when compared with the routine TSE sequence.

## Introduction

The turbo spin-echo (TSE) sequence has been widely used in musculoskeletal (MSK) imaging because of its ability to provide excellent contrast in cartilage, ligaments, menisci, and periarticular soft tissues (Kloth et al., 2014; Fritz et al., 2017; Garwood et al., 2017). However, despite the advantage of the use of multiple refocusing radiofrequency (RF) in the TSE sequence, which can effectively increase the signal-to-noise ratio (SNR) and improve the image quality, its acquisition speed is limited by T2 decay restraints and a loss of signal amplitude when using a large turbo factor (Glaser et al., 2015; Lee et al., 2015). The integrated parallel acquisition technique such as the generalized autocalibrating partially parallel acquisitions (GRAPPA) can save acquisition time through sampling only every second or third phase-encoding step, while keeping the field of view (FOV), matrix size and spatial resolution (Fritz et al., 2017, 2021). However, the number of omitted phase-encoding steps also results in the loss of SNR (Deshmane et al., 2012). In addition, aside from the SNR threshold of diagnostic image quality, the maximally possible imaging acceleration factor also depends on the geometric pattern of the coil elements and the calculated unaliasing algorithm.

Simultaneous multi-slice (SMS) imaging is a newly developed acceleration technique using multi-band RF pulses, which can simultaneously excite, acquire, and reconstruct multiple slices and readout with two-dimensional images (Larkman et al., 2001; Kenkel et al., 2016; Norbeck et al., 2018; Teruel et al., 2018). With this technique, the imaging time can be largely reduced. Furthermore, the SMS imaging technique is equipped with a blipped-Controlled Aliasing In Parallel Imaging Results In Higher Acceleration (CAIPIRINHA) (Filli et al., 2016b; Kenkel et al., 2016; Bentatou et al., 2020). Unlike the GRAPPA and compressed sensing (CS) imagining, the SMS- CAIPIRINHA not only avoids signal undersampling but also can help to reduce the g-factor penalty (Breuer et al., 2005; Setsompop et al., 2012). Thus, SMS acceleration offers a promising technique to accelerate clinical TSE protocols while preserving SNR. At present, the SMS imaging technique is mainly applied to echo planar imaging (EPI) sequences and has been used in diffusion-weighted imaging (DWI) and functional magnetic resonance (MR) imaging of the brain, breast, cardiac, liver, and pancreas (Boss et al., 2016; Yokota et al., 2017; Nazir et al., 2018; Lee et al., 2019; Machida et al., 2020). Few studies have investigated the clinical application of the SMS imaging technique in the MSK system. Benali et al. (2018) compared iPAT and SMS TSE T2-weighted imaging (T2WI) sequence in knee MRI in pediatric patients and found that SMS accelerated the T2WI sequence without impairing the diagnostic performance. Another study conducted by Fritz et al. (2017) found that fourfold–accelerated TSE through the combination of PAT2 and SMS2 enables approximately 50% shorter acquisition times compared with regular PAT2 acceleration in knee joint imaging. The previous studies have demonstrated the usefulness of SMS in knee joint imaging, however, the value of SMS in accelerating and providing expectable SNR contrast-to-noise ratio (CNR) in wrist and ankle joint have still not been investigated.

Therefore, we prospectively quantified and compared the SNR and CNR of routine TSE and SMS-TSE in the application of MSK imaging including knee, wrist and ankle joint and further compared its diagnostic performance in lesion diagnosis by using the routine TSE as standard reference.

## Materials and Methods

### Study Population

This study was approved by the institutional review board, and written informed consent was obtained from all the patients. Between January 2016 and March 2017, 79 consecutive patients with a history of hand, ankle, and knee pain or injury were included in the MR examination. Of these patients, eight were excluded because of the following exclusion criteria: (1) patients with a history of wrist (*n* = 2) or knee joint surgery (*n* = 3); (2) patients who could not tolerate the MR examination (*n* = 1); and (3) patients with severe motion artifacts that impair the MR image interpretation (*n* = 2). Finally, 71 patients, including 25 patients who underwent wrist joint MR imaging, 15 patients who underwent ankle joint MR imaging, and 31 patients underwent knee joint MR imaging, were finally enrolled.

### Magnetic Resonance Examination

For all patients, MR imaging was performed using a 3.0 T MR scanner (MAGNETOM Prisma, Siemens Healthcare, Erlangen, Germany). A 16-channel hand/wrist coil, a 15-channel knee coil and a 16-channel foot/ankle coil were used for respective examinations. The routine imaging protocol for wrist imaging included coronal T1-weighted imaging, T2WI, proton density-weighted imaging (PDWI) with fat saturation (FS), and axial T1-weighted imaging with FS. For the knee and ankle imaging, sagittal T1-weighted imaging with and without FS were also obtained. To control the comparison between the routine TSE and SMS-TSE sequences, the imaging parameters for the SMS sequences were the same (except for the axial-T2WI-FS and Cor-PDWI-FS in wrist joint imaging), except for the slice acceleration factors, which were 2 for SMS-TSE and 1 for routine TSE. The scanning time for each sequence was recorded. For the wrist joint imaging, the scanning times for the Cor-T1WI, Cor-T2WI, Cor-PDWI-FS, and axial-T2WI-FS sequences that used the routine TSE sequence were 2.55, 4.90, 4.30, and 3.17 min, respectively. Moreover, the scanning times for the SMS-TSE sequence were 1.32, 1.90, 1.70, and 2.60 min, respectively. The reason why Cor-PDWI-FS reduced about 2.5 times, and axial-T2WI-FS reduced only about 1.2 times is due to the phase over-sampling factor and repetition time setting, the echo time for routine axial-T2WI-FS were 75.0 ms and reduced to 72.0 ms with SMS-TSE sequence, and the phase over-sampling factor from 100% with routine Cor-PDWI-FS reduced 50% with SMS-TSE sequences. For the ankle joint, the scanning times for Sag-T1W, Sag-PDWI-FS, and Cor-PDWI-FS were 4.97, 5.50, and 3.50 min, respectively, and the imaging times for the SMS sequences were 2.55, 2.83, and 1.82 min, respectively. In addition, for the knee joint imaging, the scanning times for the Sag-T1W, Sag-PDWI-FS, and Cor-PDWI-FS sequences that used the routine TSE sequence were 3.15, 5.00, and 5.33 min, respectively. Moreover, the scanning times for the SMS-TSE sequence were 1.65, 2.52, and 2.70 min, respectively. The detailed parameters of the MR sequence are listed in Table 1.

**TABLE 1:** Magnetic resonance imaging parameters of the conventional and simultaneous multi-slice sequences.

Parameters	Cor-T1WI (wrist)	Cor-T2WI (wrist)	Cor-PDWI-FS (wrist/knee/ankle)	Ax-T2WI-FS (wrist)	Sag-T1WI (knee/ankle)	Sag-PDWI-FS (knee/ankle)
Repetition time (ms)	840	3,000	3,000/3,200/3,000	3,520	350/600	3,000
Echo time (ms)	11	98	53/38/30	75	13/11	34/38
Field of view (mm^2^)	230 230	230 × 230	230 × 230/160 160/159 × 159	230 × 230	160 × 160/250 × 203	160 × 160/250 × 203
Matrix	512 358	512 × 358	512 × 358/448 × 314/384 × 326	320× 224	512 × 358/576 × 403	448 314/512 × 435
Slice thickness (mm)	2.5	2.5	2.5/4/3	3	4/3	4/3
Slices	20	20	20/28/32	36	20/24	20/24
Phase oversampling	50%	100% (routine)/50% (SMS)	100%/50%/0%	30%	50%	50%
Flip angle	120°	150°	150°/120°/120°	120°	120°/135°	120°/135°
PAT mode	CAIPIRINHA (for SMS)	CAIPIRINHA (for SMS)	CAIPIRINHA (for SMS)	CAIPIRINHA (for SMS)	CAIPIRINHA (for SMS)	CAIPIRINHA (for SMS)
Phase partial Fourier	Off	Off	Off	Off	Off	Off
Acceleration factor	2 (for SMS)	2 (for SMS)	2 (for SMS)	2 (for SMS)	2 (for SMS)	2 (for SMS)
Reference scan lines	26	31	39/30/26	38	26/24	30/30
Scanning time (min)	2.55/1.32	4.90/1.90	4.30/1.70 (wrist) 5.33/2.70 (knee) 3.50/1.82 (ankle)	3.17/2.60	3.15/1.65 (knee) 4.97/2.55 (ankle)	5.00/2.52 (knee) 5.50/2.83 (ankle)

*Cor-T1WI, coronal T1-weighted imaging; Cor-T2WI, coronal T2-weighted imaging; Cor-PDWI-FS, coronal proton density-weighted imaging with fat saturation; Axial-T2WI-FS, axial T2-weighted imaging with fat saturation; Sag-T1WI, sagittal T1-weighted imaging; Sag-PDWI-FS, sagittal proton density-weighted imaging with fat saturation. For the scanning time, numbers before and after the backslash are for the routine TSE and SMS-TSE sequences.*

### Quantitative Imaging Analysis

All MR images were reviewed using a vendor supplied Syngo.*via* MR workstation. Two experienced MSK imaging radiologists reviewed the MR images and performed the quantitative measurements. The middle section was selected for the region of interest (ROI) placement. For the wrist joint measurement, a circular ROI was placed on the major thenar muscle to measure the soft tissue signal intensity (SI) of tissue [SI_(tissue)_], and the ROI was placed on the second metacarpal bone to measure the bone SI [SI_(bone)_]. For the ankle joint, the short flexor muscle and calcaneus were selected for measurement. For the knee joint, the semitendinosus and femur were selected for measurement. In addition, for the standard deviation of the background noise [SD_(background)_] measurement, the ROI was placed on the phase plane encoding direction, which was outside of the tissue but still in the FOV. The shape and size of the ROIs in the routine TSE and SMS-TSE groups were the same. Quantitative parameters of the SNR, contrast-to-noise ratio (CNR), and per-minute SNR (SNR/min) were calculated. The SNR was calculated using the following equation:

SNR=SI/(tissue)SD(background)

The CNR was calculated using the following equation:

CNR=|SI-(tissue)SI|(bone)/SD(background)

### Qualitative Imaging Analysis

For the qualitative imaging analysis, two other experienced MSK imaging radiologists who were blinded to the clinical results independently reviewed the MR images. To avoid measurement bias, all MR images were anonymized, including the patient baseline and basic MR sequence information. The MR images were randomly distributed and had no time limit on the review process. The review of routine TSE and SMS-TSE sequences in the same patient was separated by a delay period of 1–2 weeks to minimize recall. A 5-point evaluation scale system was used to determine the image quality of bone texture, cartilage, tendons, ligament, meniscus, and artifacts, and the evaluation scale system was as follows: 5 = uniform signal, excellent contrast, and no artifacts; 4 = mild signal inhomogeneity, good contrast, and mild artifacts; 3 = moderate signal inhomogeneity, moderate contrast, and moderate artifacts; 2 = strong signal inhomogeneity, poor contrast, and prominent artifacts; and 1 = non-diagnostic. When the two radiologists did not agree with each other, a consensus result was achieved. An image quality of 2 and less than 2 was excluded from further analysis. Furthermore, the imaging signs of the lesions on the SMS-TSE sequences were compared with the routine TSE sequences, which were used as the standard reference. The abnormal imaging sign of bone contusion was defined as the pattern of reticular low SI on the T1WI in marrow and high SI on T2WI-FS sequence. Other abnormal imaging signs, such as articular surface abnormality, cartilage injury, tendon degeneration or injury, and peripheral soft tissue injury, were also recorded.

### Statistical Analyses

The paired *t*-test or *Wilcoxon* signed-rank test was used to compare the SNR and CNR between the routine TSE and SMS-TSE groups in the wrist joint. The interobserver agreement between the two radiologists regarding the evaluation scale of image quality of artifacts score was determined using *the Kappa* test, with values less than 0.4, 0.4–0.6, 0.6–0.8, and greater than 0.8 indicating poor, moderate, substantial, and good agreement, respectively. For the qualitative scores of the routine TSE and SMS-TSE sequences, the *Wilcoxon signed-rank* test was used to compare differences. Furthermore, the diagnostic sensitivity, specificity, and accuracy of the SMS-TSE sequence were calculated using routine TSE sequences as a standard reference. Statistical significance was set at *p* < 0.05. All statistical analyses were performed using a statistical software package [Statistical Package for the Social Sciences (SPSS) version 17.0 (SPSS Inc., Chicago, IL, United States)].

## Results

### Imaging Time Comparison

**FIGURE 1:**
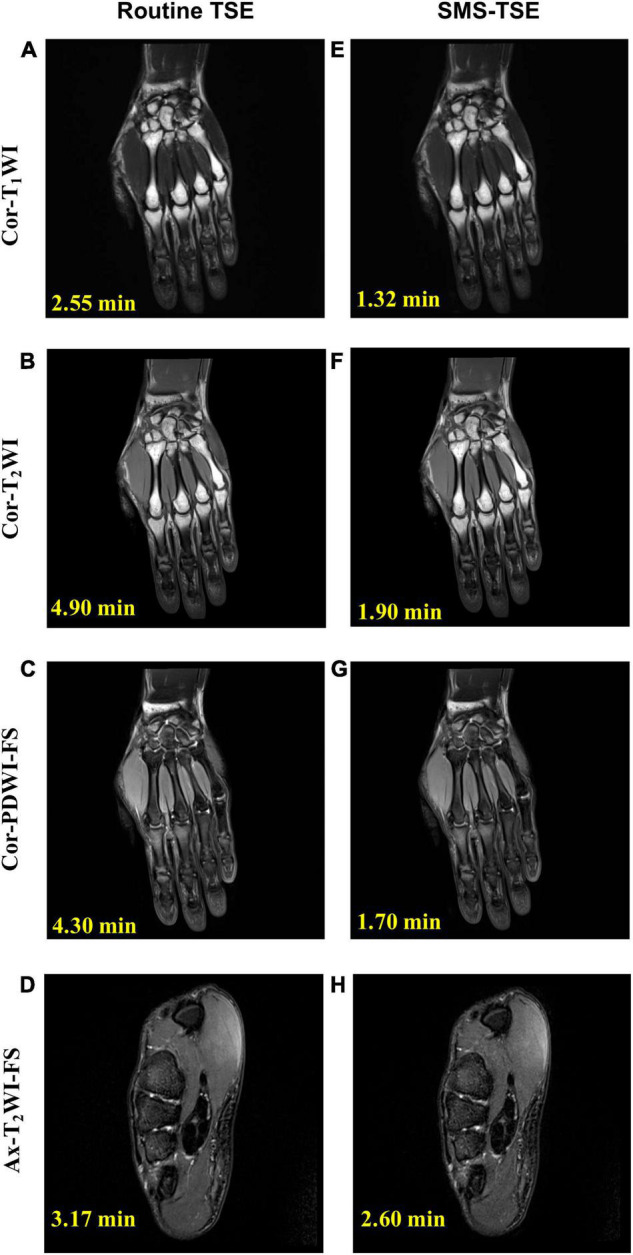
Comparison of high-resolution turbo spin-echo (TSE) sequence and simultaneous multi-slice-TSE sequence in the wrist joint imaging. The median nerve is thickened and swollen and shows hyperintensity on axial-T2-weighted imaging-fat saturation imaging (arrow), and the final diagnosis is carpal tunnel syndrome. All the images scored 5 in the image quality evaluation. For routine TSE, the SNR for Cor-T1WI **(A)**, Cor-T2WI **(B)**, Cor-PDWI-FS **(C)**, and Ax-T2WI-FS **(D)** were 228.46, 108.76, 279.91, and 123.75; and the CNR for each was 192.21, 313.92, 72.77, and 71.55. For the SMS-TSE, the SNR for Cor-T1WI **(E)**, Cor-T2WI **(F)**, Cor-PDWI-FS **(G)**, and Ax-T2WI-FS **(H)** were 208.36, 103.21, 182.86.91, and 103.71; and the CNR for each was 203.31, 308.65, 58.76, and 63.45.

**FIGURE 2:**
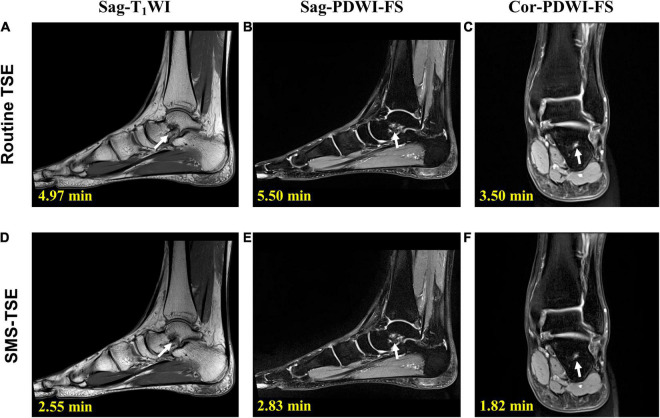
Comparison of the high-resolution turbo spin-echo (TSE) sequence and simultaneous multi-slice-TSE sequence in the ankle joint imaging. On sagittal-proton density-weighted imaging (PDWI)-fat saturation (FS) **(B,E)** and coronal-PDWI-FS **(C,F)** images, the bone marrow edema of the talus is clearly depicted (arrow). All the images scored 5 in the image quality evaluation. For routine TSE, the SNR for Sag-T1WI **(A)**, Sag-PDWI-FS **(B)** a d Cor-PDWI-FS **(C)** were 194.79, 143.01, and 158.13; and the CNR for each was 289.83, 125.38, and 147.96. For the SMS-TSE, the SNR for Sag-T1WI **(D)**, Sag-PDWI-FS **(E)** a d Cor-PDWI-FS **(F)** were 187.50, 101.80, and 147.96; and the CNR for each was 196.28, 97.76, and 123.31.

**FIGURE 3:**
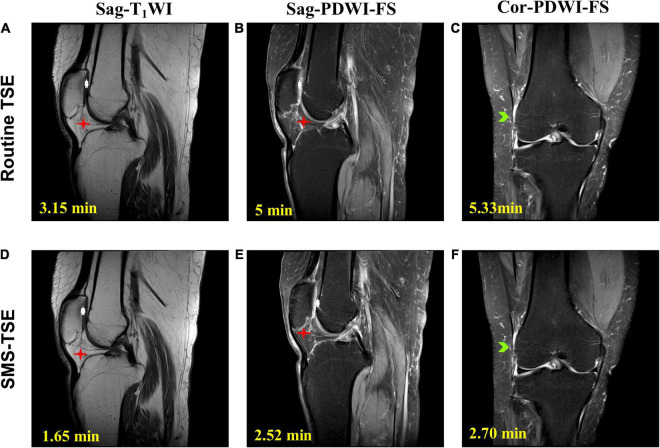
Comparison of the high-resolution turbo spin-echo (TSE) sequence and simultaneous multi-slice-TSE sequence in the knee joint imaging. On the magnetic resonance images, the degeneration of the knee joint is clearly depicted. All the images scored 5 in the image quality evaluation. For routine TSE, the SNR for Sag-T1WI **(A)**, Sag-PDWI-FS **(B)** a d Cor-PDWI-FS **(C)** were 30.75, 72.86, and 78.67; and the CNR for each was 88.89, 44.39, and 43.18. For the SMS-TSE, the SNR for Sag-T1WI **(D)**, Sag-PDWI-FS **(E)** a d Cor-PDWI-FS **(F)** were 29.22, 73.47, and 68.61; and the CNR for each was 77.11, 45.68, and 41.35.

For the wrist (Figure 1), ankle (Figure 2), and knee (Figure 3) joint imaging, the total scanning times was reduced from 14.92, 13.97, and 13.48 to 7.52 min, 7.20 and 6.84 min, thus, about 49.60, 48.46, and 49.94% total scanning time was reduced for each joint imaging.

### Quantitative Comparison of the Image Quality

**FIGURE 4:**
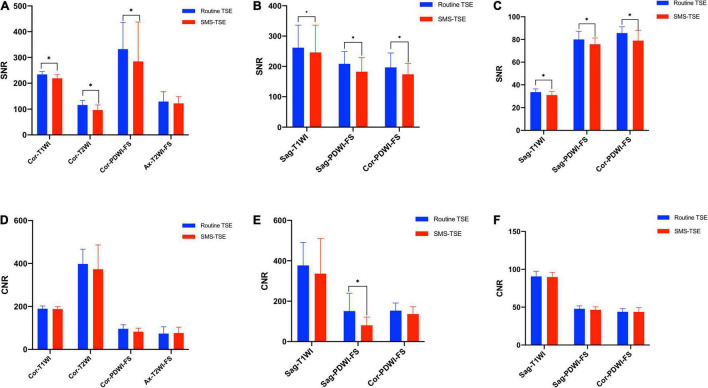
Quantitative comparison of the SNR and CNR between the high-resolution turbo spin-echo (TSE) sequence and simultaneous multi-slice-TSE imaging. The SNR of the wrist **(A)**, ankle **(B),** and knee **(C)** obtained with routine TSE sequences were significantly higher than SMS-TSE sequences except for the Cor-PDWI-FS sequence in wrist joint. No differences were obtained with CNR for the wrist **(D)**, ankle **(E)**, and knee **(F)** between the routine TSE sequences and SMS-TSE sequences except for the Sag-PDWI-FS sequence in ankle joint. “*”*p* < 0.05.

In this study, three patients were excluded. Of them, one patient was excluded because of that the patient cannot tolerant the MR examination, the other two patients were excluded as severe motion happed during the imaging. For the wrist, ankle, and knee joint imaging, the SNR of routine TSE sequences was significantly higher than that of SMS-TSE sequences (all *p* < 0.05), except for the axial-T2WI-FS (*p* = 0.296) obtained in the wrist joint. For the CNR comparison, no statistical significance was obtained from the routine TSE and SMS-TSE sequences (all *p* > 0.05). Detailed information for the comparison between the routine TSE and SMS-TSE sequences is listed in Table 2 and Figure 4.

**TABLE 2:** Comparison of the signal-to-noise ratio (SNR), contrast-to-noise ratio (CNR), and SNR/min between the routine turbo spin-echo (TSE) and simultaneous multi-slice (SMS)-TSE sequences.

Quantitative parameters	SNR		CNR	
	SMS-TSE	Routine TSE	SMS-TSE	Routine TSE
**Wrist joint**				
Cor-T1WI	219.10 ± 14.00^*‡^	234.16 ± 11.20	187.81 ± 11.10^‡^	189.75 ± 12.69
Cor-T2WI	96.56 ± 19.34^*†^	115.44 ± 17.46	373.31 ± 113.18^†^	398.07 ± 68.37
Cor-PDWI-FS	284.75 ± 152.97^†*^	332.40 ± 104.04	82.68 ± 16.54^‡^	96.01 ± 19.20
Axial-T2WI-FS	121.69 ± 26.14^†^	128.89 ± 37.87	76.36 ± 27.24^‡^	73.76 ± 31.91
**Ankle joint**				
Sag-T1WI	246.01 ± 90.03^*‡^	261.71 ± 74.13	336.21 ± 173.56^‡^	376.99 ± 113.72
Sag-PDWI-FS	182.28 ± 46.58^*‡^	208.33 ± 40.63	80.47 ± 40.81^*‡^	150.98 ± 88.27
Cor-PDWI-FS	173.76 ± 35.53^*‡^	197.76 ± 47.45	136.34 ± 36.20^‡^	153.32 ± 37.69
**Knee joint**				
Sag-T1W	30.96 ± 3.13^*‡^	33.58 ± 2.84	89.75 ± 6.12^†^	90.65 ± 6.73
Sag-PDWI-FS	75.76 ± 5.56^*‡^	79.95 ± 7.22	46.45 ± 3.96^‡^	47.92 ± 3.85
Cor-PDWI-FS	78.96 ± 9.13^*†^	85.66 ± 5.55	43.78 ± 5.62^†^	43.93 ± 4.11

“*”p < 0.05; Cor-T1WI, coronal T1-weighted imaging; Cor-T2WI, coronal T2-weighted imaging; Cor-PDWI-FS, coronal proton density-weighted imaging with fat saturation; Axial-T2WI-FS, axial T2-weighted imaging with fat saturation; Sag-T1WI, sagittal T1-weighted imaging; Sag-PDWI-FS, sagittal proton density-weighted imaging with fat saturation. “‡” Difference was tested by using paired t-test. “†” Difference was tested by using Wilcoxon signed-rank test.

### Qualitative Comparison of the Image Quality

Substantial to good interobserver agreement was obtained for the routine TSE [Kappa*_(wrist)_* = 0.821, Kappa*_(ankle)_* = 0.765, Kappa*_(knee)_* = 0.767] and SMS-TSE [Kappa*_(wrist)_* = 0.839, Kappa*_(ankle)_* = 0.745, Kappa*_(knee)_* = 0.736] sequences for the motion artifact score evaluation. For the wrist joint, the display of bone texture obtained from Cor-T2w and Cor-PDWI-FS of SMS-TSE was lower than that of routine TSE sequence (all *p* < 0.05), whereas for the display of cartilage and tendons, the difference was also obtained from the Cor-PDWI-FS sequence (all *p* < 0.05) and axial-T2WI-FS [*P*_(tendon)_< 0.05]. However, no statistically significant differences were obtained from other sequences (all *p* > 0.05). For the knee joint, the display of cartilage and ligament obtained from the SMS-TSE sequences was significantly lower than that of routine TSE sequences (all *p* < 0.05). However, for the comparison of the motion artifact score, no significant difference was observed between the routine TSE and SMS-TSE sequences (all *p* > 0.05). Additionally, for the display of the ankle joint, no statistically significant differences were observed in the bone texture, cartilage, ligaments, and artifacts between the routine and SMS sequences (all *p* > 0.05). Detailed information about these evaluation scales is presented in Table 3 and Supplementary Material.

**TABLE 3:** Qualitative comparison of the evaluation score between the routine turbo spin-echo (TSE) and simultaneous multi-slice (SMS)-TSE sequences.

Qualitative parameters	SMS-TSE						Routine TSE					
	Cor-T1WI	Cor-T2WI	Ax-T2WI-FS	Cor-PDWI-FS	Sag-T1WI	Sag-PDWI-FS	Cor-T1WI	Cor-T2WI	Axial-T2WI-FS	Cor-PDWI-FS	Sag- T1WI	Sag-PDWI-FS
**Wrist joint**												
Bone texture	3.98 ± 0.76	3.94 ± 0.75*	4.24 ± 0.76	3.98 ± 0.76*	…	…	4.10 ± 0.71	4.10 ± 0.71	4.38 ± 0.71	4.10 ± 0.83	…	…
Cartilage	3.96 ± 0.75	4.12 ± 0.65	4.08 ± 0.72	4.04 ± 0.73*	…	…	4.04 ± 0.75	4.18 ± 0.61	4.18 ± 0.69	4.20 ± 0.65	…	…
Tendon	4.08 ± 0.79	4.04 ± 0.75	3.98 ± 0.70	4.04 ± 0.85*	…	…	4.12 ± 0.79	4.20 ± 0.72	3.94 ± 0.75	4.20 ± 0.78	…	…
Artifact	3.94 ± 0.73	4.02 ± 0.73	4.04 ± 0.69	3.94 ± 0.78	…	…	4.02 ± 0.70	4.10 ± 0.69	4.02 ± 0.73	4.00 ± 0.75	…	…
**Ankle joint**												
Bone texture	…	…	…	3.97 ± 0.69	3.93 ± 0.84	3.80 ± 0.75	…	…	…	4.03 ± 0.77	4.17 ± 0.79	3.97 ± 0.79
Cartilage	…	…	…	3.80 ± 0.75	3.87 ± 0.74	4.03 ± 0.69	…	…	…	4.07 ± 0.70	3.80 ± 0.70	4.10 ± 0.63
Ligament	…	…	…	3.87 ± 0.83	3.90 ± 0.87	3.80 ± 0.77	…	…	…	3.93 ± 0.80	3.93 ± 0.86	4.00 ± 0.73
Artifact	…	…	…	3.83 ± 0.75	3.87 ± 0.74	3.83 ± 0.72	…	…	…	3.87 ± 0.77	3.87 ± 0.72	3.96 ± 0.77
**Knee joint**												
Bone texture	…	…	…	3.97 ± 0.77	4.31 ± 0.73	3.95 ± 0.76*	…	…	…	4.06 ± 0.83	4.44 ± 0.63	4.16 ± 0.71
Cartilage	…	…	…	3.87 ± 0.68*	3.82 ± 0.71*	3.98 ± 0.65*	…	…	…	4.18 ± 0.63	4.03 ± 0.68	4.13 ± 0.69
Ligament	…	…	…	4.03 ± 0.82*	3.94 ± 0.76*	3.89 ± 0.68*	…	…	…	4.18 ± 0.80	4.11 ± 0.78	4.19 ± 0.68
Meniscus	…	…	…	4.02 ± 0.83*	4.11 ± 0.68	4.02 ± 0.82*	…	…	…	4.21 ± 0.83	4.19 ± 0.70	4.24 ± 0.74
Artifact	…	…	…	3.82 ± 0.73	3.76 ± 0.66*	3.94 ± 0.72*	…	…	…	3.92 ± 0.68	3.94 ± 0.69	4.06 ± 0.69

“*”p < 0.05; Cor-T1w, coronal T1-weighted imaging; Cor-T2w, coronal T2-weighted imaging; Cor-PDWI-FS, coronal proton density-weighted imaging with fat saturation; Axial-T2WI-FS, axial T2-weighted imaging with fat saturation; Sag-T1W, sagittal T1-weighted imaging; Sag-PDWI-F, sagittal proton density-weighted imaging with fat saturation.

### Diagnostic Performance

For wrist imaging, one patient (4.0%, 1/25) who was diagnosed with triangular fibrocartilage complex (TFCC) injury and one patient (4.0%, 1/25) diagnosed with wrist joint degeneration using the routine TSE sequences were diagnosed as having normal wrists using the SMS-TSE sequence. Thus, the diagnostic sensitivity, specificity, and accuracy for the SMS-TSE sequence were 88.24% (15/17), 100% (8/8), and 92% (23/25), respectively. For ankle imaging, only one patient (6.7%, 1/15) who was diagnosed as normal ankle joint using routine TSE sequence was diagnosed as degenerative ankle joint using SMS-TSE sequence, and the diagnostic sensitivity, specificity, and accuracy for the SMS-TSE sequence were 100% (11/11), 75% (3/4), and 93.33% (14/15), respectively. Additionally, for knee imaging, two patients (6.5%, 2/31) who were diagnosed with knee joint degeneration and one patient (3.2%, 1/31) diagnosed with bone marrow edema using the routine TSE sequences were diagnosed as having normal knees using the SMS-TSE sequence. Thus, the diagnostic sensitivity, specificity, and accuracy for the SMS-TSE sequence of the knee joint were 87.50% (21/24), 85.71% (6/7), and 87.10% (27/31), respectively. Detailed information about the diagnostic performance is listed in Table 4.

**TABLE 4:** Comparison of the diagnostic performance of wrist, ankle, and knee joints between the routine turbo spin-echo (TSE) and simultaneous multi-slice (SMS)-TSE sequence.

Parameters	Sensitivity (%, n)	Specificity (%, n)	Accuracy (%, n)	PPV (%, n)	NPV (%, n)
**Wrist joint**					
Wrist degeneration	87.5 (7/8)	100 (17/17)	96.0 (24/25)	0 (0/17)	12.5 (1/8)
TFCC injury	66.67 (2/3)	100 (22/22)	96.0 (24/25)	0 (0/22)	33.3 (1/3)
Lunate necrosis	100 (2/2)	100 (23/23)	100 (25/25)	0 (0/23)	0 (0/2)
CTS	100 (1/1)	100 (24/24)	100 (25/25)	0 (0/24)	0 (0/1)
PVS	100 (1/1)	100 (24/24)	100 (25/25)	0 (0/24)	0 (0/1)
Ganglion cyst	100 (2/2)	100 (23/23)	100 (25/25)	0 (0/23)	0 (0/2)
Normal wrist joint	100 (8/8)	100 (17/17)	100 (25/25)	0 (0/17)	0 (0/8)
**Ankle joint**					
Knee degeneration	100 (5/5)	90 (9/10)	93.33 (14/15)	10 (1/10)	0 (0/5)
Distal fibular fracture	100 (2/2)	100 (13/13)	100 (15/15)	0 (0/13)	0 (0/2)
Joint effusion	100 (3/3)	100 (12/12)	100 (15/15)	0 (0/12)	0 (0/3)
Osteochondritis dissecans	100 (1/1)	100 (14/14)	100 (15/15)	0 (0/14)	0 (0/1)
Normal ankle	75 (3/4)	100 (11/11)	93.33 (14/15)	0 (0/11)	25 (1/4)
**Knee joint**					
Knee degeneration	86.7 (13/15)	100 (16/16)	93.6 (29/31)	0 (0/16)	13.3 (2/15)
Meniscal injury	100 (2/2)	100 (29/29)	100 (31/31)	0 (0/29)	0 (0/2)
Bone marrow edema	66.7 (2/3)	3.7 (1/27)	93.6 (29/31)	3.6 (1/28)	33.3 (1/3)
Cruciate ligament injury	100 (3/3)	100 (28/28)	100 (31/31)	0 (0/28)	0 (0/3)
Soft tissue injury	100 (1/1)	100 (30/30)	100 (31/31)	0 (0/30)	0 (0/1)
Normal knee joint	85.71 (6/7)	100 (24/24)	100 (31/31)	0 (0/24)	14.29 (1/7)

*TFCC, triangular fibrocartilage complex; CTS, carpal tunnel syndrome; PVS, pigmented villonodular synovitis; PPV, positive predictive value; NPV, negative predictive value.*

## Discussion

In the present study, the difference in the imaging time and quality between the routine TSE and SMS-TSE sequences was compared, and the diagnostic performance of the SMS-TSE sequence was evaluated. The results demonstrated that when compared with the routine TSE sequence, the SMS-TSE sequence can significantly lower the imaging time, with only little loss of the SNR and that may have slightly impact on diagnostic performance. However, no difference was observed in the CNR between the SMS and SMS-TSE groups, but the tSNR/min of the SMS-TSE group was significantly higher than that of the SMS group.

Our results indicated that the imaging time using the SMS-TSE sequence was significantly shorter than that using the routine TSE sequence. A previous study conducted by Haraikawa et al. (2018) found that the use of SMS-TSE sequence in the hip joint can reduce the total acquisition time of the three sequences from 12 min and 16 s to 7 min and 35 s. Fritz et al. (2017) found that the scanning time can achieve 50% shorter through the combination of PAT2 and SMS2 compared with regular PAT2 acceleration in knee joint imaging. Benali et al. (2018) also found a scanning time reduction from 5:08 to 3:02 min in pediatric knee imaging, and almost a 49% reduction of time was achieved. Additionally, other study conducted by Filli et al. (2016a) investigated the use of SMS in the application of breast DWI, and the results demonstrated that when combined with SMS technique, the imaging time for readout-segmented EPI (rs-EPI) was greatly reduced, as the imaging time for routine rs-EPI was 4:21 min, and when the slice accelerations were 2 and 3, the imaging times were 2 min and 35 s and 1 min and 44 s, respectively. In this study, we found that 49.60% of scanning time was lower using the SMS-TSE protocol compared with the routine TSE sequence. The reason why SMS can greatly reduce the imaging time can be explained by the fact that SMS is a fast imaging technique that can simultaneously excite several slices using multi-band RF, which can reduce the imaging time.

It is noteworthy that for the comparison of CNR between the routine TSE and SMS-TSE sequences, no significant difference was obtained from the wrist and knee joints (all *p* > 0.05), and differences were only obtained from the SNR measurement. Benali et al. (2018) also found a 30% lower SNR of the SMS sequence compared to the conventional sequence. This result is consistent with our results. Another study conducted by Fritz et al. (2017) also showed that with the increasing SMS acceleration factors, the SNR decreased. However, the difference was that during the acquisition the GRAPPA reference lines were integrated as part of the SMS scan in the Fritz study, but a separate scan strategy of the CAIPIRINHA method was adopted in this study. SNR reduction in SMS-TSE images may be because we used a small slice gap (10%) in the SMS TSE sequence to match the conventional TSE sequence, which is not enough to avoid the crosstalk effect of slices and cause the signal reduction in SMS case (Fritz et al., 2017). Using of a larger slice gaps and use of radiofrequency pulses with improved slice profile will further help to mitigate this effect. Besides, no parallel acquisition technology is used in conventional TSE, so there’s no g-factor penalty theoretically. Thus, a balance between the imaging speed and image quality is warranted. Additionally, it is noteworthy that some false positive or negative cases were still depicted by using the SMS-TSE sequence despite the similar CNR between these two sequences. Jung et al. (2012) assessed the diagnostic performance of shoulder MR arthrography with 3D isotropic fat-suppressed (FS) turbo spin-echo sequence (TSE-SPACE) for supraspinatus tendon tears in comparison with 2D conventional sequences at 3.0 T, their results found that the diagnostic sensitivity and specificity on 2D images were only numerical higher than 3D images but no statistical differences were obtained, and the false interpretations on 3D TSE-SPACE in their study were largely attributable to the blurring effect. Another study conducted by Kijowski et al. (2009) compared a three-dimensional isotropic resolution fast spin-echo sequence (FSE-Cube) with a routine magnetic resonance (MR) imaging protocol in knee joint imaging, their results found that the FSE-Cube had significantly higher sensitivity (*P* = 0.039) but significantly lower specificity (*P* = 0.003) than the routine MR imaging protocol for detecting cartilage lesions, and that may due to the FSE-Cube images had thinner section thicknesses than the 2D FSE images, with no gaps between sections, this likely provided better visibility of small cartilage lesions. In this study, the reason why false positive or negative cases were depicted when use the routine TSE as standard may be related with the random scan caused variability such as the shift slice position relative to anatomy or lower image quality in SMS-TSE sequence in some of the cases. As the image artifact and quality degradation appeared in some of the SMS-TSE images, thus, small lesions on SMS-TSE images might be neglected.

For the qualitative comparison of SMS-TSE and routine TSE sequences, the display of bone texture, cartilage, and tendon in some TSE sequences were superior to the SMS-TSE sequence, and the lesion detection capability of the routine TSE sequence was also superior to that of the SMS-TSE sequence. Furthermore, for wrist imaging, one patient diagnosed with wrist degeneration and another patient diagnosed with TFCC injury in routine TSE sequences were missed using the SMS-TSE sequence. This might be explained by the fact that the routine TSE sequence entails a higher SNR, which may enable a clearer display of the normal anatomical structures and lesion detection.

More importantly, some other acceleration techniques have also been used in the muscleskeletal (MSK) system. Compressed sensing (CS) is a newer technique that takes advantage of the inherent redundancy and compressibility of MR images to undersample k-space, allowing fast acquisition at higher acceleration factors (Matcuk et al., 2020). Matcuk et al. (2020) showed that CS can be applied to different MRI knee sequences and imaging planes with time savings ranging from 36 s to 2 min and 58 s (Matcuk et al., 2020). Other study conducted by Iuga et al. (2020) also found that CS can significantly decrease (34.39% for 2D CS 2 and 54.17% for 3D CS 10) scan time in knee imaging with unchanged diagnostic certainty and overall image impression compared to the clinical reference. Furthermore, deep learning (DL) a new acceleration strategy has also been used in MSK imaging. Herrmann et al. (2021) reported that DL image reconstruction for TSE sequences in MSK imaging is feasible, enabling a remarkable time saving (up to 75%), whilst maintaining excellent image quality and diagnostic confidence. Other advanced MRI techniques like synthetic MR imaging have also been used in MSK system, and which may provide unprecedented opportunities for rapid musculoskeletal imaging while retaining quality, comprehensiveness, and diagnostic performance (Fritz et al., 2021).

### Limitation

Our study has some limitations. First, the study sample was relatively small, which may leave the question about the reproducibility open; thus, we should continue to enroll more patients. Second, the head-to-head comparison of another fast imaging technique, like GRAPPA was not conducted in this study; however, a previous study has shown that compared with the GRAPPA technique, the SMS accelerated the T2WI sequence without impairing the diagnostic performance (Benali et al., 2018). Finally, different slice acceleration factors were not selected in our study, as higher slice acceleration factors may lead to faster imaging, but the specific absorption rate (SAR) also increased. In this study, a slice acceleration factor of 2 was selected because under this acceleration factor, the SAR is still within a reasonable range and may not be harmful to the body.

## Conclusion

The use of the SMS-TSE sequence in the wrist, ankle, and knee joints can significantly reduce the scanning time and show similar image quality when compared with the routine TSE sequence. Additionally, given the increasing number of MR examinations, the time savings by applying SMS to the routinely obtained MRI sequences as part of a MSK imaging could be used to increase patient throughput.

## Data Availability Statement

The original contributions presented in the study are included in the article/Supplementary Material, further inquiries can be directed to the corresponding author/s.

## Ethics Statement

The studies involving human participants were reviewed and approved by the Henan Provincial People’s Hospital. The patients/participants provided their written informed consent to participate in this study.

## Author Contributions

YG designed and supervised the study, including data collection and analysis. FG, ZW, and SD performed most of the investigations, including data collection and analysis, and wrote the manuscript. XK and SW provided guidance for data analysis. All authors have read and approved the manuscript.

## Conflict of Interest

The authors declare that the research was conducted in the absence of any commercial or financial relationships that could be construed as a potential conflict of interest.

## Publisher’s Note

All claims expressed in this article are solely those of the authors and do not necessarily represent those of their affiliated organizations, or those of the publisher, the editors and the reviewers. Any product that may be evaluated in this article, or claim that may be made by its manufacturer, is not guaranteed or endorsed by the publisher.
