# Supplementary material for: High-Resolution Simultaneous Multi-Slice Accelerated Turbo Spin-Echo Musculoskeletal Imaging: A Head-to-Head Comparison With Routine Turbo Spin-Echo Imaging
Source: Front Physiol. 2021 Dec 21;12:759888. doi: 10.3389/fphys.2021.759888 (PMC8724040; doi:10.3389/fphys.2021.759888)
Supplement: Supplementary file 1 [file Data_Sheet_1.PDF]

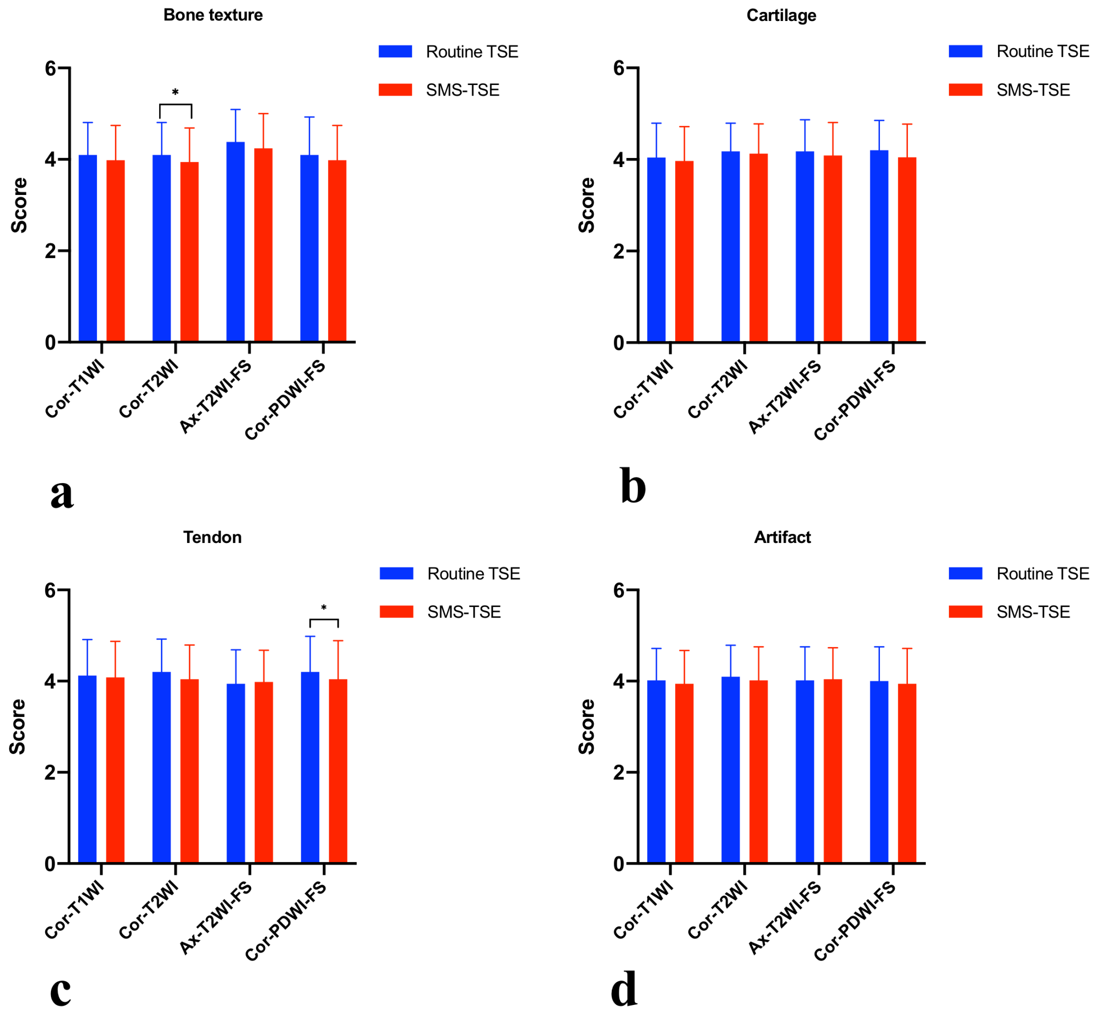

Supplement Figure 1: Qualitative comparison of the image quality in bone texture (a), cartilage (b), tendon (c) and artifact (d) in wrist joint between routine TSE and SMS-TSE sequences. Differences were obtained in bone texture with Cor-T2WI and tendon with Cor-PDWI-FS sequence. “*, $p<0.05$”

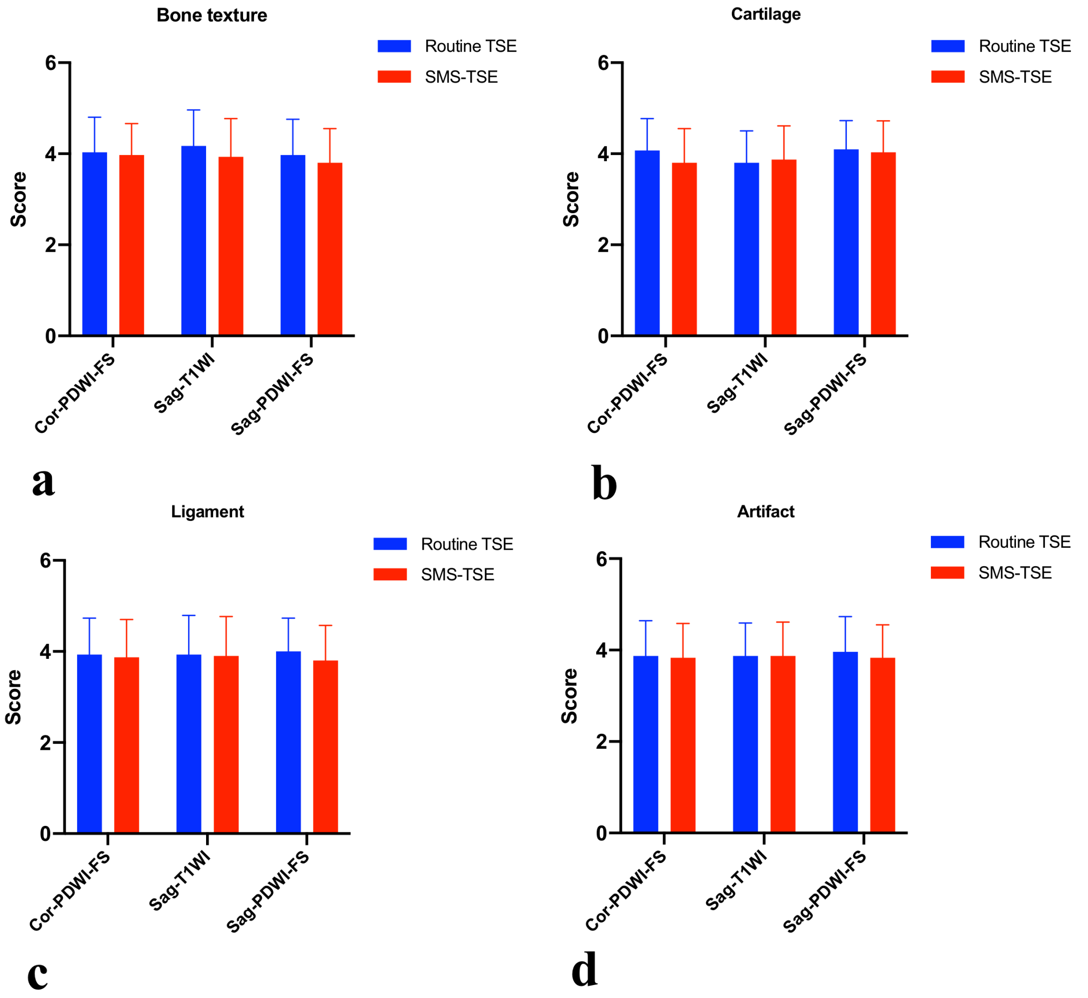

Supplement Figure 2: Qualitative comparison of the image quality in bone texture (a), cartilage (b), ligament (c) and artifact (d) in ankle joint between routine TSE and SMS-TSE sequences.

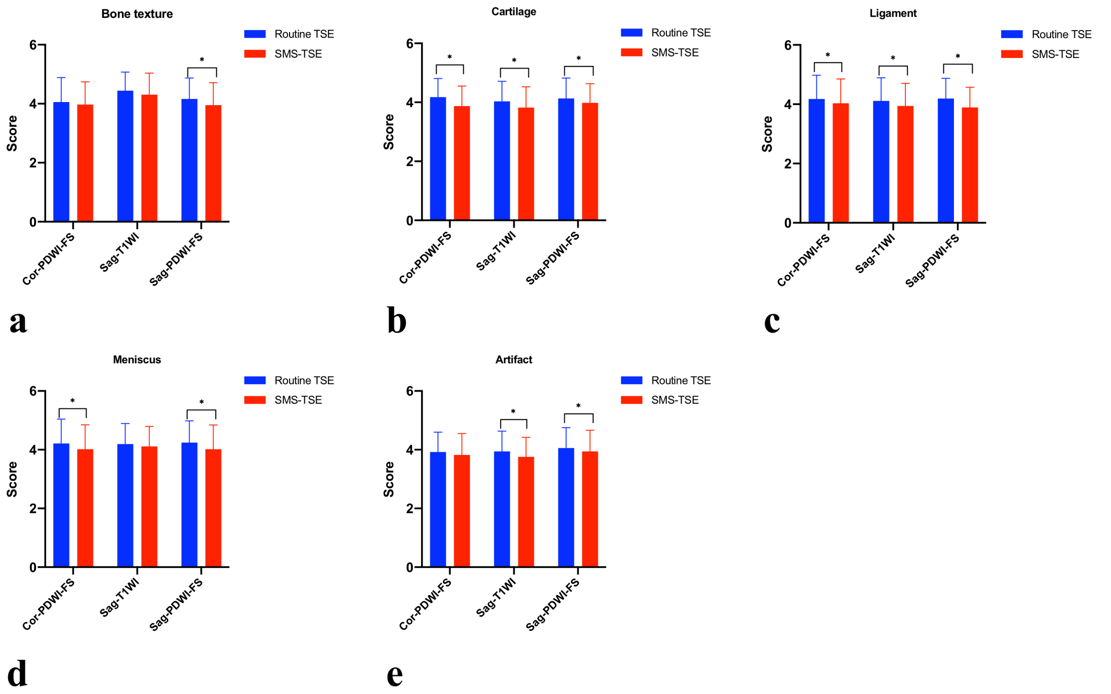

Supplement Figure 3: Qualitative comparison of the image quality in bone texture (a), cartilage (b), ligament (c), meniscus (d) and artifact (e) in knee joint between routine TSE and SMS-TSE sequences. “*, $p<0.05$”

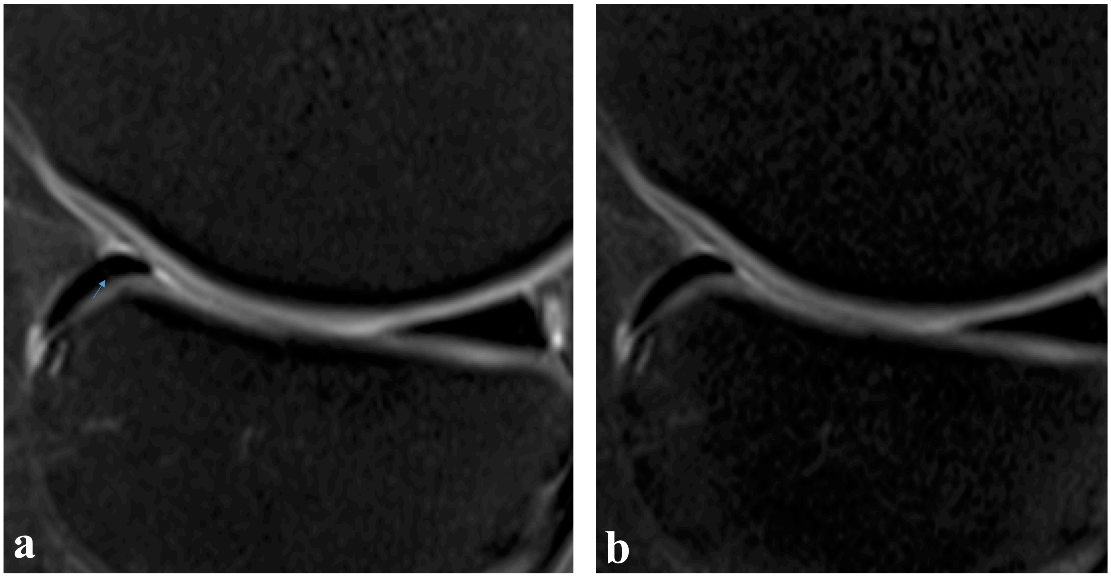

Supplement Figure 4: Comparison of the routine TSE (a) and SMS-TSE image (b) in knee joint. On the routine TSE (a), a light white line which mean the line tear of the anterior meniscus can be seen(arrow), while no white line can be seen in the SMS-TSE image.
